# Newborn hearing screening: outcomes and management of infants

**DOI:** 10.1590/1806-9282.20251993

**Published:** 2026-06-15

**Authors:** Hatice Kübra Bozkurt, Fatma Ceyda Akın Öçal, Ercan Karababa, Ayşe Nur Balaban, Rumeysa Nur Akbaş, Öykü Özbaş Kes, Emine Aydın

**Affiliations:** 1 University of Health Science, Gülhane Faculty of Health Science, Department of Audiology – Ankara, Türkiye.; 2 University of Health Science, Gülhane Training and Research Hospital, Department of Otorhinolaryngology – Ankara, Türkiye.

**Keywords:** Hearing loss, Newborn, Risk factors, Auditory brainstem response

## Abstract

**OBJECTIVE:**

The aim of this study was to evaluate the prevalence and characteristics of hearing loss among infants referred after newborn hearing screening in a tertiary referral center in Türkiye and to examine its association with demographic and clinical risk factors.

**METHODS:**

A retrospective study was conducted at SBÜ Gülhane Training and Research Hospital between January 2021 and December 2024, including 1,322 infants referred after newborn hearing screening due to either a "refer" result or the presence of risk factors despite passing automated auditory brainstem response. Relevant risk factors were analyzed.

**RESULTS:**

Hearing loss was detected in 92 infants (7.0%), including 3.3% with unilateral and 3.7% with bilateral loss. By severity, the distribution was mild (2.8%), moderate (0.8%), moderately severe (0.3%), and profound (1.5%). While most infants with unilateral loss did not receive amplification, bilateral cases showed higher rates of hearing aid (28.6%) and cochlear implant (14.3%) use. Family history of hearing loss was the only factor significantly associated with device adoption (p<0.05).

**CONCLUSION:**

The prevalence of hearing loss in referred infants was higher than in population-based studies and aligned with findings in high-risk groups. Differences in device use between unilateral and bilateral cases highlight the importance of parental counseling. Strengthening follow-up and family awareness is crucial to improving early intervention outcomes, particularly in infants without a family history.

## INTRODUCTION

Hearing loss is a common health problem affecting millions of people worldwide^1^. If left untreated, it can impact many aspects of life, delaying speech and language development in children and increasing the risk of cognitive decline and dementia in older adults. It is also closely associated with reduced academic achievement, limited employment opportunities, and emotional problems such as loneliness, depression, and anxiety^2^. Auditory rehabilitation can mitigate many of these negative consequences, particularly when hearing loss is identified at birth or shortly after onset^3^. Universal newborn hearing screening (UNHS) plays a critical role in early diagnosis and intervention, preventing long-term adverse outcomes^1^. UNHS is recommended for the early detection of congenital hearing loss^4^. In Türkiye, the National Newborn Hearing Screening Program was initiated in 2003, adopting the "1-3-6 model" proposed by the Joint Committee on Infant Hearing, which includes screening by 1 month, diagnosis by 3 months, and intervention by 6 months^5,6^.

Worldwide, screening protocols and preventive strategies used in newborn hearing screening programs vary not only across countries but also within regions of the same country^7^.

Automated objective auditory screening methods, such as automated auditory brainstem response (aABR) and automated otoacoustic emissions (aOAE), are practical and reliable but have certain limitations. The literature emphasizes that these devices are insufficient in detecting mild hearing loss and that the threshold-detection capacity of aABR is limited to approximately 40–45 dB. Moreover, aOAE has been reported to fail in detecting auditory neuropathy^5,8^. In Türkiye, newborn hearing screening initially used a combined aOAE and aABR protocol, but since 2020, only aABR has been applied nationwide^5^.

While most previous studies in Türkiye have reported the screening outcomes of all newborns^9-11^, this study aims to provide a unique perspective by focusing on infants referred for further evaluation, representing a high-risk group. In addition, it aims to determine the prevalence of hearing loss in a state hospital located in the capital of Türkiye, which also receives a high volume of referrals from surrounding provinces, to examine its association with demographic factors, and to provide evidence-based contributions to policies and practices for improving hearing health.

## METHODS

### Study design and procedure

This hospital-based cross-sectional study was conducted at SBÜ Gülhane Training and Research Hospital, a referral center located in the capital of Türkiye, Ankara. It is based on file reviews of infants born between January 2021 and December 2024 who were directed to this center following newborn hearing screening. The study included 1,322 infants: those with risk factors for hearing loss despite receiving a "pass" result in the initial screening, as well as those with a "refer" result. All infants were first examined by an otorhinolaryngologist before further evaluation. Babies referred due to risk factors underwent tympanometric assessment and aABR testing, while those who failed the initial screening received a comprehensive audiological evaluation, including tympanometry and diagnostic ABR. Infants diagnosed with hearing loss were subsequently referred back to the otorhinolaryngologist for advanced evaluation and treatment planning.

Ethical approval for the study was obtained from the Non-Interventional Research Ethics Committee of SBÜ Gülhane Training and Research Hospital (Decision No: 2025/76). Due to the retrospective nature of the study, informed consent was not obtained. The collected data included risk factors such as the infant's sex, mode of delivery, maternal febrile illness during pregnancy, family history of congenital hearing loss, consanguinity, maternal diseases, admission to intensive care (>5 days), prematurity (<37 weeks), low birth weight (<1,500 g), hyperbilirubinemia, craniofacial anomalies, and the use of ototoxic drugs. In addition, final hearing outcomes, established diagnoses, and the interventions or treatments applied were also included in the dataset.

### Statistical analysis

The data were analyzed using the SPSS for Windows version 22 software package. For data analysis, numbers, percentages, minimum and maximum values, means, and standard deviations were calculated, and the chi-square test was used for the comparison of categorical variables^12^. In the study, only infants diagnosed with sensorineural hearing loss were evaluated in relation to risk factors. Infants with conductive or mixed-type hearing loss were not included in the analysis due to the insufficient number of cases for statistical evaluation.

## RESULTS

### Participants

Of the participants, 589 (44.6%) were female and 733 (55.4%) were male, with a total of 1,322 infants included in the study. Among them, 383 (29.0%) were delivered vaginally, while 939 (71.0%) were delivered by cesarean section. The most common risk factors for hearing loss observed among the participants were admission to the neonatal intensive care unit (64.9%), prematurity (23.1%), and febrile illness (19.1%). Other factors are presented in detail in Table 1.

**Table 1:** Patient demographics and risk factors.

		n	%
Gender	Female	589	44.6
	Male	733	55.4
Type of delivery	Vaginal	383	29.0
	Cesarean	939	71.0
Risk factors for hearing loss	Febrile illness	252	19.1
	Family history of congenital hearing loss	95	7.2
	Consanguineous marriage	68	5.1
	Maternal diseases	194	14.7
	Stay in intensive care (>5 days)	858	64.9
	Premature	305	23.1
	Low birth weight (<1,500 g)	102	7.7
	Bilirubin >20 mg/dL	178	13.5
	Craniofacial anomalies	9	0.7
	Ototoxic drug	32	2.4

Values are given as number (%).

### Analyses

In the screening ABR test, 1,166 infants (88.2%) passed in the right ear and 1,162 (87.9%) in the left ear. A total of 156 infants (11.8%) failed in the right ear and 160 (12.1%) in the left ear. Following detailed evaluation, hearing loss was identified in 43 infants with unilateral loss (3.3%) and in 49 with bilateral loss (3.7%), resulting in a total of 92 infants (141 ears) diagnosed with hearing loss. In 1,230 participants (93.0%), no hearing loss was detected. Regarding the degree of hearing loss, the most common findings were mild and profound losses. Among the data obtained from 1,322 infants (2,644 ears), the distribution of hearing loss was as follows: mild (2.8%, 73 ears), moderate (0.8%, 22 ears), moderately severe (0.3%, 7 ears), and profound (1.5%, 39 ears).

In terms of intervention/treatment methods, the majority of infants with hearing loss (67.4%) did not use any device; 20.7% used a hearing aid, 7.6% a cochlear implant, and in 4.3% of cases, the information was unknown (Table 2). Intervention methods differed according to whether the hearing loss was unilateral or bilateral. While 82.9% of unilateral cases did not use a device and only 12.2% used a hearing aid, in bilateral cases, 53.1% did not use a device, 28.6% used a hearing aid, and 14.3% used a cochlear implant. The distribution of intervention/treatment methods according to demographic and clinical characteristics is presented in Table 3, and a statistically significant higher rate of hearing aid use was found only among infants with a family history of congenital hearing loss (p<0.05). No significant differences were observed for other variables (p>0.05).

**Table 2:** Results of automated auditory brainstem response and diagnostic evaluation for all infants.

					n	%
aABR test result		Right ear		Pass	1.166	88.2
				Refer	156	11.8
		Left ear		Pass	1.162	87.9
				Refer	160	12.1
Hearing status/diagnosis		Unilateral hearing loss			43	3.3
		Bilateral hearing loss			49	3.7
		Normal hearing			1.230	93.0
Degree of hearing loss	Total n=92	Right ear	Mild		36	50.0
			Moderate		11	15.3
			Moderately severe		5	6.9
			Profound		20	27.8
		Left ear	Mild		37	53.6
			Moderate		11	16.0
			Moderately severe		2	2.9
			Profound		19	27.5
	Unilateral n=43	Right ear n=23	Mild		12	52.2
			Moderate		1	4.3
			Moderately severe		2	8.7
			Profound		8	34.8
		Left ear n=20	Mild		9	45.0
			Moderate		3	15.0
			Moderately severe		2	10.0
			Profound		6	30.0
	Bilateral n=49	Right ear n=49	Mild		24	49.0
			Moderate		10	20.4
			Moderately severe		3	6.1
			Profound		12	24.5
		Left ear n=49	Mild		28	57.1
			Moderate		8	16.3
			Moderately severe		0	0.0
			Profound		13	26.6
Intervention/treatment		No intervention			62	67.4
		Hearing aid			19	20.7
		Cochlear implant			7	7.6
		None			4	4.3

Values are given as number (%). aABR: automated auditory brainstem response.

**Table 3:** Comparison of treatment approaches according to demographic characteristics and risk factors in patients diagnosed with hearing loss.

		No intervention		Hearing aid		Cochlear implant		None		Statistical significance
		n	%	n	%	n	%	n	%	
Gender	Female	25	41.7	9	47.4	2	28.6	3	75.0	χ^2^=2.449 p=0.485
	Male	35	58.3	10	52.6	5	71.4	1	25.0	
Type of delivery	Vaginal	22	36.7	4	21.1	3	42.9	3	75.0	χ^2^=4.665 p=0.199
	Cesarean	38	63.3	15	78.9	4	57.1	1	25.0	
Febrile illness	Absent	57	95.0	16	84.2	7	100	4	100	χ^2^=3.595 p=0.309
	Present	3	5.0	3	15.8	0	0.0	0	0.0	
Family history of congenital hearing loss	Absent	57	95.0	13	68.4	6	85.7	4	100	χ^2^=10.899 **p=0.012**
	Present	3	5.0	6	31.6	1	14.3	0	0.0	
Consanguineous marriage	Absent	52	86.7	17	89.5	7	100	3	75.0	χ^2^=1.703 p=0.636
	Present	8	13.3	2	10.5	0	0.0	1	25.0	
Maternal diseases	Absent	59	98.3	19	100	7	100	4	100	χ^2^=0.506 p=0.918
	Present	1	1.7	0	0.0	0	0.0	0	0.0	
Stay in intensive care (>5 days)	Absent	34	56.7	12	63.2	5	71.4	3	75.0	χ^2^=1.113 p=0.774
	Present	26	43.3	7	36.8	2	28.6	1	25.0	
Premature	Absent	53	88.3	15	78.9	7	100	4	100	χ^2^=2.930 p=0.403
	Present	7	11.7	4	21.1	0	0.0	0	0.0	
Low birth weight (<1,500 g)	Absent	57	95.0	16	84.2	7	100	4	100	χ^2^=3.595 p=0.309
	Present	3	5.0	3	15.8	0	0.0	0	0.0	
Bilirubin >20 mg/dL	Absent	54	90.0	18	94.7	7	100.0	3	75.0	χ^2^=2.365 p=0.500
	Present	6	10.0	1	5.3	0	0.0	1	25.0	
Craniofacial anomalies	Absent	60	100	19	100	7	100	4	100	–
	Present	–	–	–	–	–	–	–	–	
Ototoxic drug	Absent	59	98.3	19	100	7	100.0	4	100	χ^2^=0.506 p=0.918
	Present	1	1.7	0	0.0	0	0.0	0	0.0	

As Fisher's exact chi-square test was applied, the chi-square statistic is not reported. χ^2^: chi-square analysis. p<0.05 was considered statistically significant. Bold values indicate statistically significant results.

## DISCUSSION

We present the audiological evaluation results of 1,322 infants who were referred to SBÜ Gülhane Training and Research Hospital following newborn hearing screening between 2021 and 2024. In this retrospective review, the rate of hearing loss was found to be 7%, which is considerably higher than the prevalence rates reported in the general population within the scope of UNHS (1–2/1,000 in high-income countries, 2–6/1,000 in middle-income countries, and 19–53/1,000 in low-income countries)^13,14^. The main reason for this difference is that our study does not reflect community-based prevalence but rather includes infants who were referred after receiving a "refer" result in the screening, as well as those who obtained a "pass" result in the initial test but were referred due to the presence of risk factors. In this context, the observed rate is more consistent with studies conducted in high-risk groups and demonstrates that referred infants carry a substantially higher risk of hearing loss^7,10,15-18^.

When the distribution by degree of hearing loss was examined, the rate of profound loss in our study was found to be 1.5%. This result is similar to the reported rate of 1.9% for bilateral severe/profound congenital hearing loss among infants monitored in neonatal intensive care units^17^. Our findings indicate that among referred infants, not only mild and moderate but also clinically significant severe losses were observed at a notable rate.

In our study, treatment methods were observed to differ according to whether the hearing loss was unilateral or bilateral. In unilateral cases, the majority of infants did not use a device despite the presence of hearing loss, whereas in bilateral cases, the rates of hearing aid and cochlear implant use were higher. Similarly, the literature also reports lower intervention rates in unilateral hearing loss, with families showing less inclination toward device use, particularly in cases of mild loss^19-21^. In the study by Fitzpatrick et al., children with mild bilateral hearing loss were found to be nearly seven times more likely to use their hearing aids regularly compared to those with unilateral loss^19^. Likewise, Walker et al. reported that children with mild hearing loss used their hearing aids less frequently and less consistently compared to those with severe hearing loss^20^. Our findings are consistent with these results. It is considered that the predominance of mild cases among unilateral losses and the presence of normal hearing in the contralateral ear reduce families’ inclination toward device use.

Our study demonstrated that a family history of hearing loss was an important factor increasing the likelihood of device use, whereas other risk factors did not have a significant influence on device use preferences. The literature also indicates that parents with a family history of hearing loss have greater awareness of the consequences of hearing impairment^22,23^. Furthermore, evidence suggests that the involvement of family members in the hearing health process facilitates decisions regarding amplification. Similarly, qualitative research has shown that parents with a family history of hearing loss are more familiar with the process and act more promptly during diagnosis^24^. In light of our findings, it is considered that this increased awareness may also facilitate amplification decisions. This highlights the importance of early awareness and referral in cases with a positive family history, while in cases without such history, awareness-raising and counseling services appear to be even more critical.

Newborn hearing screening programs have made it possible to identify mild-to-moderate or unilateral hearing losses—conditions that in the past were often not diagnosed until later childhood—already during infancy^3^. Our study supports this observation. In conclusion, hearing loss remains a prevalent and growing public health concern. Policies and health services must therefore prioritize early diagnosis, prevention, and treatment strategies, particularly in populations at risk.

## Data Availability

The datasets generated and/or analyzed during the current study are available from the corresponding author upon reasonable request.
